# Machine learning for oral frailty factors in hospitalized schizophrenia patients: two-stage feature selection and SHAP analysis

**DOI:** 10.3389/fpsyt.2026.1802742

**Published:** 2026-04-20

**Authors:** Yue Fu, Xing Yang, Tao Zhang, Yingying Wang, Shihan Tang, Zheng Luo, Cui Yang, Dongmei Wu

**Affiliations:** 1 School of Nursing, Chengdu Medical College, Chengdu, China; 2 Department of Nursing, Chengdu University of Traditional Chinese Medicine, Chengdu, China; 3 Tianfu College of Southwest University of Finance and Economics, Chengdu, China; 4 Department of Emergency, Zigong Fourth People’s Hospital, Zigong, China; 5 Department of Nursing, Sichuan Provincial People’s Hospital, School of Medicine, University of Electronic Science and Technology of China, Chengdu, China

**Keywords:** feature selection, machine learning, oral frailty, schizophrenia, SHAP

## Abstract

**Background:**

Long-term hospitalized patients with schizophrenia (SZ) often experience significant oral health problems, and oral frailty (OF) can further exacerbate the decline in their quality of life. However, the status and key influencing factors contributing to OF in this population remain insufficiently explored. Most existing studies rely on traditional regression models, which are prone to overfitting when processing high-dimensional data, making accurate risk identification difficult. This study aims to clarify the current status of OF in this population in Southwest China, identify the influencing factors, and optimize the predictive model using machine learning (ML), thereby providing a basis for clinical practice.

**Methods:**

A total of 404 long-term hospitalized patients with SZ from three psychiatric hospitals in Southwest China were enrolled in this study. The Oral Frailty Index-8 was employed to assess OF. Nine feature selection methods and five ML models were employed to optimize the model through two-stage feature selection, while Shapley Additive Explanations (SHAP) were used to analyze the model’s predictive logic.

**Results:**

The prevalence of OF in this population was determined to be 69.3%. The optimal model identified was the random forest, with the Area Under the Curve increasing to 0.779 following two-stage optimization. Compared to non-feature selection, performance improved by approximately 6.57%. SHAP analysis revealed that the Number of Teeth, Number of Psychiatric Hospitalizations, Self-discontinuation of Medication, Marital Status, and Age were core risk factors for OF.

**Conclusion:**

The prevalence of OF in long-term hospitalized patients with SZ is notably high. Two-stage feature selection enhances the accuracy of the predictive model, and the identified core factors can serve as a reference for developing individualized oral intervention programs in clinical practice.

## 1 Introduction

Schizophrenia (SZ) is a severe and chronic psychiatric disorder characterized by positive symptoms, negative symptoms, and cognitive impairments, with a lifetime prevalence of approximately 1% (1–3). SZ significantly impairs patients’ social functioning and activities of daily living and is closely associated with multiple physical comorbidities (4–7). Oral health problems are particularly prevalent among patients with SZ. Epidemiological studies indicate that patients with SZ are at a higher risk for oral health problems and exhibit significantly poorer oral conditions compared with the general population (8–10). These problems are even more pronounced among hospitalized SZ patients due to environmental constraints, persistent negative symptoms, and reduced self-care capacity (11, 12). The etiology of oral health problems in SZ patients is multifactorial. First, disease-related cognitive impairments and negative symptoms significantly compromise patients’ ability to maintain oral hygiene (13). In addition, the anticholinergic side effects of antipsychotic medications can lead to xerostomia, impairing oral mucosal protection and salivary cleansing functions (4, 14). Furthermore, limited access to dental care and poor adherence to oral treatments further increase the risk of oral diseases (15). Traditional oral health assessments, which often focus on isolated functional aspects, fail to capture the complex interplay between oral and systemic health in SZ patients, underscoring the need for a more comprehensive evaluation approach.

Oral frailty (OF) is an emerging concept in oral medicine, defined as a comprehensive condition involving structural abnormalities and multidimensional declines in oral function, often accompanied by deterioration in physical, cognitive, and social functioning (16). OF provides a holistic framework for assessing oral health and poses multidimensional risks, particularly for hospitalized patients with SZ. Physically, OF can lead to dietary dysfunction, malnutrition, and sarcopenia (17). Psychologically, OF may contribute to late-life cognitive impairment and depression (18). More critically, OF has been associated with several life-threatening adverse outcomes. As an independent risk factor for falls, OF can lead to severe trauma such as fractures, thereby posing a direct threat to patients’ physical health (19). Functionally, OF is associated with an increased risk of dysphagia (20). Notably, the incidence of swallowing disorders among hospitalized SZ patients is 31%, which is 6% and 25% higher than that in non-hospitalized SZ patients and the general population, respectively (17). Dysphagia increases the risk of aspiration, potentially resulting in pulmonary infections (21). Additionally, oral health problems are a common cause of airway obstruction in SZ patients, which is the third leading cause of death among hospitalized individuals with SZ (22). Therefore, systematically identifying the determinants of OF in hospitalized SZ patients is essential for developing targeted intervention strategies.

Early intervention is fundamental to managing non-communicable chronic diseases, and the precise identification of risk factors is crucial to designing effective interventions (23). Despite the significant prognostic value of OF in hospitalized SZ patients, substantial research gaps persist in this field. First, existing studies have not reached a consensus regarding OF risk factors, and there is a notable lack of systematic investigations into the prevalence and specific determinants of OF in this high-risk group. Second, current research predominantly relies on traditional regression models. Although these methods can validate predefined hypotheses, they are prone to overfitting when dealing with high-dimensional or highly correlated variables, limiting their ability to accurately capture complex relationships between risk factors and outcomes (24–26). These limitations render traditional methods insufficient for precise risk assessment.

Machine learning (ML), an important branch of artificial intelligence, integrates methodologies from statistics, probability theory, and computer science, and has emerged as a promising approach to overcome these limitations (27). ML iteratively learns patterns from large datasets without assumptions of linearity or variable independence (28). It enables prediction and analysis through nonlinear modeling and complex interactions while facilitating the identification of key predictors through feature selection, thus offering an effective approach to decipher intricate variable relationships (29, 30). In psychiatry, ML has been shown to improve diagnostic accuracy and prognostic prediction, providing evidence-based support for treatment selection (29). However, the clinical utility of ML models is often limited by their “black-box” nature (26). To address this limitation, we applied Shapley Additive Explanations (SHAP), an interpretability method that translates model predictions into clinically interpretable feature contributions, thus bridging the gap between complex models and clinical decision-making.

In summary, long-term hospitalized patients with SZ face substantial oral health challenges. However, research on OF in this population is limited, and existing methods often fail to accurately identify key determinants. Therefore, this study focuses on long-term hospitalized SZ patients and employs a two-stage ML feature se-lection approach to assess the prevalence of OF and identify its critical determinants. In addition, SHAP was applied to interpret the contribution of each predictor, providing evidence-based support for the precise identification of high-risk individuals and the development of targeted clinical interventions.

## 2 Methods

### 2.1 Study participants

This cross-sectional study was conducted between November 2024 and March 2025 using a convenience sampling method. Long-term hospitalized patients with SZ were recruited from three psychiatric specialty hospitals in Southwest China, including two tertiary (Grade III Level A) hospitals and one secondary (Grade II Level A) hospital. Written informed consent was obtained from all participants prior to inclusion in the study. This study adhered to the STROBE guideline. The study protocol was reviewed and approved by the Institutional Ethics Review Committee (Approval No [2024]. Ethics Review No. 88).

#### 2.1.1 Inclusion and exclusion criteria

Inclusion criteria were: (1) age ≥ 18 years; (2) current long-term hospitalization, defined as a single hospitalization exceeding 60 days or cumulative annual hospitalization exceeding 90 days; (3) diagnosis of SZ based on the International Classification of Diseases, Tenth Revision (ICD-10); (4) clinically stable during the survey period, defined as a Positive and Negative Syndrome Scale score < 60; and (5) no severe cognitive, visual, auditory, or communication impairments. Exclusion criteria included: (1) comorbid psychiatric disorders other than SZ as classified in ICD-10; and (2) severe physical illnesses or organic brain disorders that prevented participation. A total of 430 questionnaires were distributed, of which 404 were valid, resulting in a response rate of 93.95%.

### 2.2 Survey tools

#### 2.2.1 General information questionnaire

A self-designed general information questionnaire was developed based on a literature review and expert consultation. The first section collects sociodemographic data, including age, gender, marital status, pre-admission residence, smoking status, alcohol consumption, visits per month, and sedentary time. The second section includes disease-related characteristics, such as number of chronic conditions, disease duration, medication type, number of psychiatric hospitalizations, self-discontinuation of medication, number of teeth, and dental caries.

#### 2.2.2 Oral frailty index 8

The Oral Frailty Index-8 (OFI-8), developed by Tanaka et al (31), was used to assess OF. The Chinese version was cross-culturally adapted by Chen et al (32) using Brislin’s translation and back-translation procedure. It consists of five dimensions with eight items, yielding a total score ranging from 0 to 11. A score of ≥4 indicates OF. The scale showed good internal consistency, with a Cronbach’s *α* coefficient of 0.713.

#### 2.2.3 Calgary depression scale for schizophrenia

The Calgary Depression Scale for Schizophrenia (CDSS), developed by Addington et al (33), was employed. The Chinese version was translated by Zhang et al (34), and its reliability and validity were verified by Zhou et al (35) The scale includes nine items, with scores ranging from 0 to 27. A score of ≥6 signifies significant depressive symptoms. The scale exhibited good reliability and validity, with a Cronbach’s α coefficient of 0.805.

#### 2.2.4 Barthel index

The Barthel Index (BI), developed by Mahoney and Barthel (36), is used to evaluate an individual’s ability to perform activities of daily living (ADLs). The Chinese version was validated by Hou et al (37) The instrument consists of 10 items with a total score ranging from 0 to 100, with score ≤40 indicating severe depend-ency. The scale exhibits strong reliability and validity, with a Cronbach’s *α* coefficient of 0.916.

### 2.3 Statistical analysis

**Figure 1:**
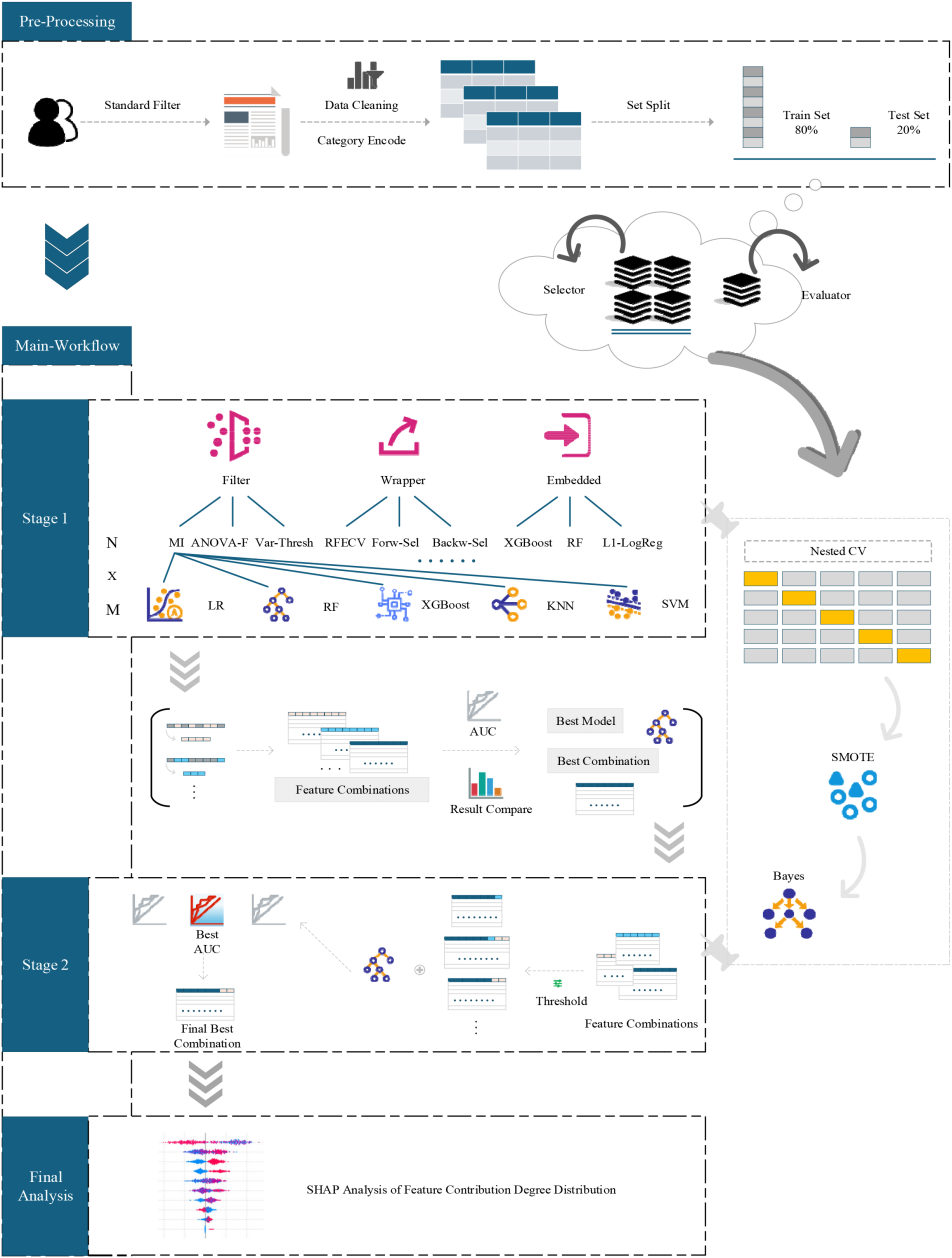
Research framework of the two-stage feature selection and machine learning analysis.

All statistical analyses were conducted using Python version 3.12.7. The core analysis libraries included scikit-learn (version 1.7.0) for model construction and feature selection, XGBoost (version 3.0.2) for gradient boosting, matplotlib (version 3.10.3) for visualization, and SHAP (version 0.48.0) for interpretability analysis. The overall analytical workflow is visualized in **Figure 1**.

#### 2.3.1 Data preprocessing

Data preprocessing was performed in accordance with established ML protocols. Missing values were imputed according to variable type: continuous variables with the median and categorical variables with the mode. The target variable OFI-8 was dichotomized, with scores ≥4 classified as OF and scores <4 as non-OF. Feature variables consisted of five continuous variables (age, disease duration, number of teeth, Number of Current Comorbidities, and number of psychiatric hospitalizations) and 13 categorical variables dichotomized into binary format (BI, gender, alcohol consumption, CDSS, self-discontinuation of medication, education level, Visits per Month, medication type, smoking status, marital status, pre-admission residence, and dental caries). Prior to model training, continuous variables were standardized, and categorical variables were binary encoded as 0/1 based on predefined rules. The dataset was randomly partitioned into training and test sets using stratified sampling (80/20 split), followed by tests to ensure no significant distributional differences in the target variable. Feature selection procedures were performed exclusively on the training set, while the test set was held out from feature selection to prevent information leakage. This approach ensured that feature selection was based solely on training data, reducing the risk of overfitting and ensuring reliable validation on the independent test set.

#### 2.3.2 Feature selection methods

To systematically evaluate the effect of feature selection on model performance, this study applied nine feature selection methods categorized into three standard types of machine learning feature selection approaches: filter methods, wrapper methods, and embedded methods. Filter methods refer to feature screening approaches that select variables based on statistical indicators independent of model training, wrapper methods are feature selection strategies that optimize feature subsets by evaluating model performance through cross-validation, and embedded methods represent feature selection techniques integrated directly into the model training process with built-in feature importance calculation. The selection criteria and applicable scenarios for each individual method are summarized in **Table 1**.

**Table 1:** Classification of feature selection methods.

Category	Method name	Selection criterion	Applicable scenario
Filter Methods	Variance Thresholding	Features with low variance contribute little to modeling	Rapid dimensionality reduction for high-dimensional data
	ANOVA F-Value	F-value measures the linear relationship between features and the target	Continuous variable selection
	Mutual Information	Captures non-linear relationships between features and the target	Small-sample non-linear data
Wrapper Methods	Recursive Feature Elimination with Cross-Validation (RFECV)	Recursively optimizes feature subsets via cross-validation	Medium-scale data
	Forward Selection	Gradually adds features with the highest contribution	Significant interaction effects between features
	Backward Elimination	Gradually removes redundant features with low contribution	Moderate number of features
Embedded Methods	L1 Regularized Logistic Regression	L1 regularization generates sparse solutions (eliminating irrelevant features)	Interpretability requirements for high-dimensional data
	Random Forest	Evaluates feature importance via Gini impurity	Non-linear relationship modeling
	XGBoost	Optimizes feature selection via gain importance	Complex pattern recognition

#### 2.3.3 Model construction and evaluation

To comprehensively evaluate the performance of different models in predicting OF, this study employed five classical algorithms: Logistic Regression (LR), Support Vector Machine (SVM), Random Forest (RF), eXtreme Gradient Boosting (XGBoost), and K-Nearest Neighbors (KNN). To mitigate class imbalance, the Synthetic Minority Over-sampling Technique (SMOTE) was employed to augment minority class samples, improving the model’s ability to identify minority instances. Model evaluation was performed using a 5-fold cross-validation strategy with three repetitions, with the Area Under the Curve (AUC) serving as the primary metric. Hyperparameter tuning was carried out using Bayesian optimization, with the parameter search space defined by the default scikit-learn settings. Model performance was evaluated based on the mean AUC value to ensure robust results.

#### 2.3.4 Two-stage optimization strategy

This study employed a two-stage feature selection optimization strategy: The first stage focused on feature selection and preliminary optimization evaluation, combining 50 combinations derived from unselected features, nine feature selection methods, and five classification models to evaluate and identify the optimal feature selection method, the corresponding classification model, and the preliminary optimal feature subset. The second stage emphasized model optimization and feature fusion validation, excluding the category of the optimal method identified in the first stage. Grid search was used to extract common features from the remaining methods, forming three types of candidate subsets: (1) the union of the preliminary optimal subset and single-category common features; (2) the union of intersections between the preliminary optimal subset and multi-category common features; and (3) a control group comprising only the preliminary optimal subset. Finally, Bayesian hyperparameter optimization was applied to validate the performance of candidate subsets on the test set, with the optimal feature subset determined based on the highest AUC value.

#### 2.3.5 Interpretability analysis

SHAP was adopted in this research to implement interpretability analysis. Monte Carlo sampling (n = 1000) was utilized to compute the SHAP values of individual features, so as to guarantee the robustness of the estimation results. Afterwards, a global feature importance map was constructed to quantify the contribution degrees of pivotal features, and the non-linear correlations between these features and OF were revealed according to the distribution pattern of feature contributions.

## 3 Results

### 3.1 General characteristics of the study sample

The final study sample comprised 404 long-term hospitalized SZ patients, with an OF prevalence of 69.3%. The sample included 251 males (62.1%) and 153 females (37.9%). The median age was 57 years, and the median disease duration was 27 years. A comparative analysis of general characteristics between the training and test sets revealed no statistically significant differences (all P > 0.05), as detailed in **Table 2**.

**Table 2:** Comparison of general characteristics between training set and test set​.

Feature	Level	Original set (n = 404)	Training set (n = 323)	Test set (n = 81)	P-value
Age	Median (IQR*)	57 (52-62)	56 (51-62)	58 (52-61)	0.427
Gender	Male	251 (62.1%)	201 (62.2%)	50 (61.7%)	0.999
	Female	153 (37.9%)	122 (37.8%)	31 (38.3%)	
Marital Status	Married	239 (59.2%)	192 (59.4%)	47 (58.0%)	0.916
	Unmarried	165 (40.8%)	131 (40.6%)	34 (42.0%)	
Pre-admission Residence	Urban	320 (79.2%)	251 (77.7%)	69 (85.2%)	0.184
	Rural	84 (20.8%)	72 (22.3%)	12 (14.8%)	
Education Level	Higher Education (College and above)	116 (28.7%)	89 (27.6%)	27 (33.3%)	0.373
	Non-higher Education (High school and below)	288 (71.3%)	234 (72.4%)	54 (66.7%)	
Smoking Status	No	275 (68.1%)	218 (67.5%)	57 (70.4%)	0.716
	Yes	129 (31.9%)	105 (32.5%)	24 (29.6%)	
Alcohol Consumption	No	395 (97.8%)	315 (97.5%)	80 (98.8%)	0.798
	Yes	9 (2.2%)	8 (2.5%)	1 (1.2%)	
Visits per Month	>1 Time	76 (18.8%)	60 (18.6%)	16 (19.8%)	0.934
	≤1 Time	328 (81.2%)	263 (81.4%)	65 (80.2%)	
Number of Current Comorbidities	Median (IQR*)	1 (0-2)	1 (0-2)	1 (0-2)	0.762
​​Disease Duration	Median (IQR*)	27 (19-34)	28 (19-34)	27 (21-33)	0.884
​​Medication Type	≤1 Category	41 (10.1%)	32 (9.9%)	9 (11.1%)	0.908
	>1 Category	363 (89.9%)	291 (90.1%)	72 (88.9%)	
Number of Psychiatric Hospitalizations	Median (IQR*)	27 (14-36)	27 (13-36)	27 (19-37)	0.342
Self-discontinuation of Medication	No	173 (42.8%)	137 (42.4%)	36 (44.4%)	0.838
	Yes	231 (57.2%)	186 (57.6%)	45 (55.6%)	
Sedentary Time	<6 Hours	191 (47.3%)	152 (47.1%)	39 (48.1%)	0.959
	≥6 Hours	213 (52.7%)	171 (52.9%)	42 (51.9%)	
Number of Teeth	Median (IQR*)	25 (12-30)	25 (12-30)	26 (13-30)	0.817
Dental Caries	Absent	76 (18.8%)	64 (19.8%)	12 (14.8%)	0.384
	Present	328 (81.2%)	259 (80.2%)	69 (85.2%)	
BI	No Functional Impairment	132 (32.7%)	102 (31.6%)	30 (37.0%)	0.421
	With Functional Impairment	272 (67.3%)	221 (68.4%)	51 (63.0%)	
CDSS	No Depression	387 (95.8%)	311 (96.3%)	76 (93.8%)	0.499
	With Depression	17 (4.2%)	12 (3.7%)	5 (6.2%)	
OFI-8	No OF	124 (30.7%)	99 (30.7%)	25 (30.9%)	0.999
	OF	280 (69.3%)	224 (69.3%)	56 (69.1%)	

*IQR, Interquartile Range. Continuous variables were compared using the Mann-Whitney U test; categorical variables were compared using the Chi-square test.

### 3.2 Feature selection results

**Figure 2:**
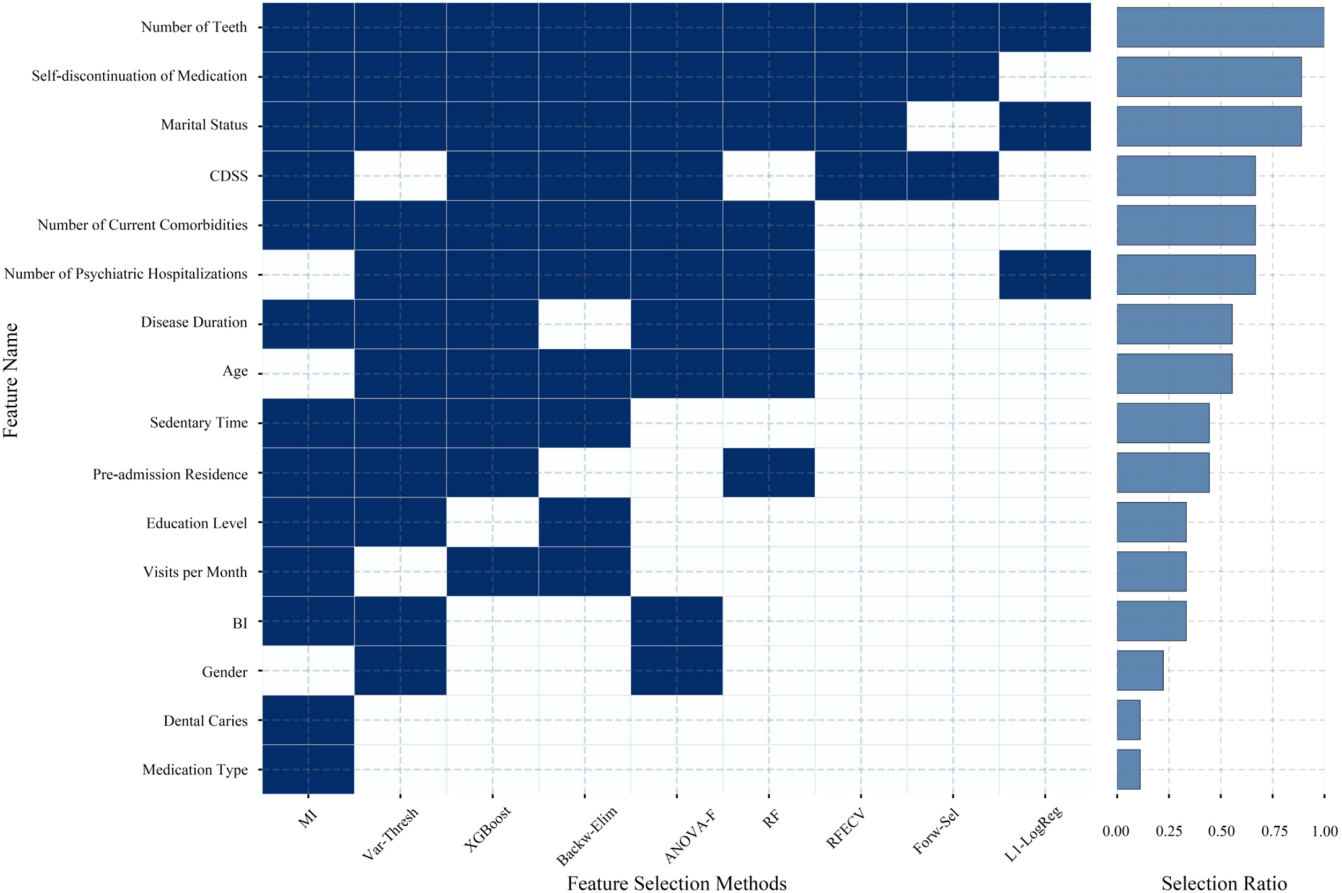
Feature distribution following stage I feature selection. Alcohol consumption and smoking status were not selected by any of the methods, and thus are not displayed.

**Figure 2** depicts the selection outcomes of 18 candidate features across the nine feature selection methods in Stage I. The number of retained features varies across different categories of methods: Filter Methods retain an average of 11.7 features, Wrapper Methods retain an average of 5.7 features, and Embedded Methods retain an average of 7.3 features. Furthermore, the consistency of feature selection also exhibits differences: Number of Teeth is retained by all methods; Self-discontinuation of Medication, Marital Status, CDSS, Number of Current Comorbidities, Number of Psychiatric Hospitalizations, Disease Duration, and Age are selected by most methods. In contrast, Gender, Dental Caries, and Medication Type are selected by only a few methods, while Alcohol Consumption and Smoking Status are not selected by any method.

**Figure 3:**
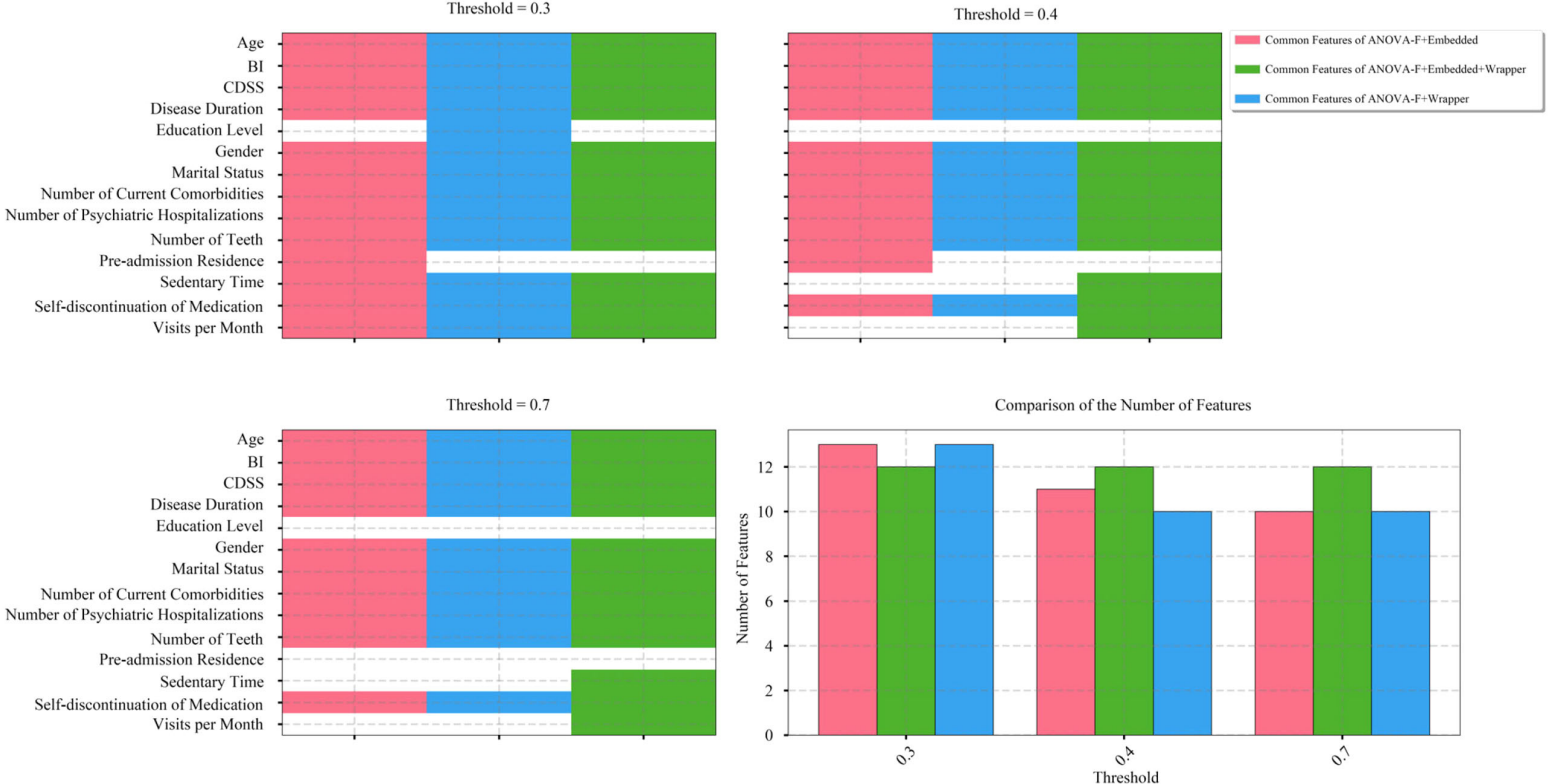
Comparison of feature combinations across different thresholds in Stage II feature selection. The feature selection results under other thresholds are consistent with those under 0.3/0.4/0.7, and thus are not displayed.

Based on the ANOVA F-Value (identified as the optimal feature selection method in Stage I), several new feature combinations were generated by filtering combinations from other categories using varying thresholds and merging them with the optimal combination identified in Stage I. **Figure 3** displays the feature selection status across diverse feature combinations at different threshold values in Stage II. Among these features, Age, BI, CDSS, Disease Duration, Gender, Marital Status, Number of Current Comorbidities, Number of Psychiatric Hospitalizations, Number of Teeth, and Self-discontinuation of Medication were consistently selected across all feature combinations, regardless of the threshold range. Additionally, the “Common Features of ANOVA-F + Wrapper + Filter” combination consistently retained 12 features across all threshold values.

### 3.3 Model performance comparison

**Figure 4:**
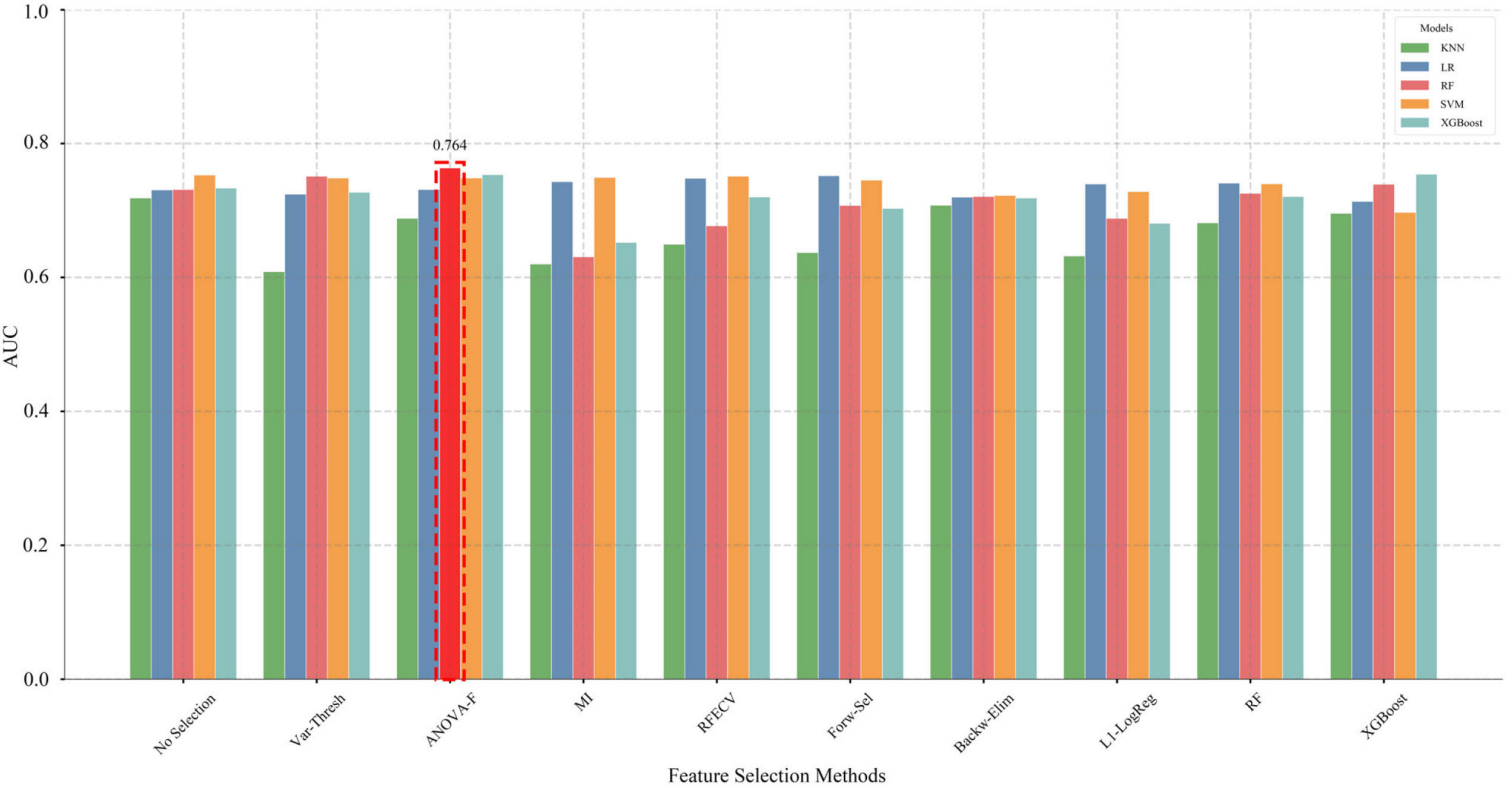
Model performance comparison following stage I feature selection.

**Figure 4** summarizes the comparative AUC performance of different feature selection methods paired with five classification models in Stage I. In the non-feature selection group, the SVM model achieved the highest performance (AUC = 0.753), while the KNN model exhibited the lowest performance (AUC = 0.719). After implementing feature selection, most models showed varying degrees of AUC improvement. Notably, the “ANOVA-F + Random Forest” combination achieved the optimal performance, with an AUC of 0.764, reflecting a 4.51% improvement compared to the original Random Forest model without feature selection (AUC = 0.731).

**Figure 5:**
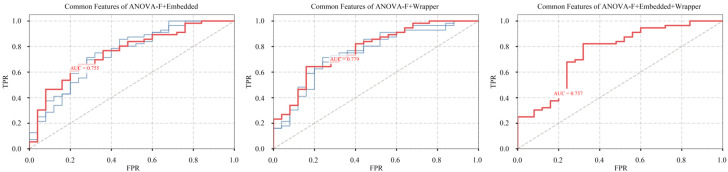
ROC curve comparison for stage II feature optimization.

Building on the optimal feature combination identified in Stage I, this stage employs Random Forest as the final classification model to evaluate the performance improvements of expanded feature subsets under different thresholds. As visualized in **Figure 5**, the integration of features selected from other methodological categories further improves model predictive performance. Specifically, the fusion of common features identified through Bagging increased the AUC of the new feature combination to 0.779, reflecting a 1.96% improvement over the optimal model from Stage I and a 6.57% increase compared to the original model without feature selection.

### 3.4 Interpretability analysis

**Figure 6:**
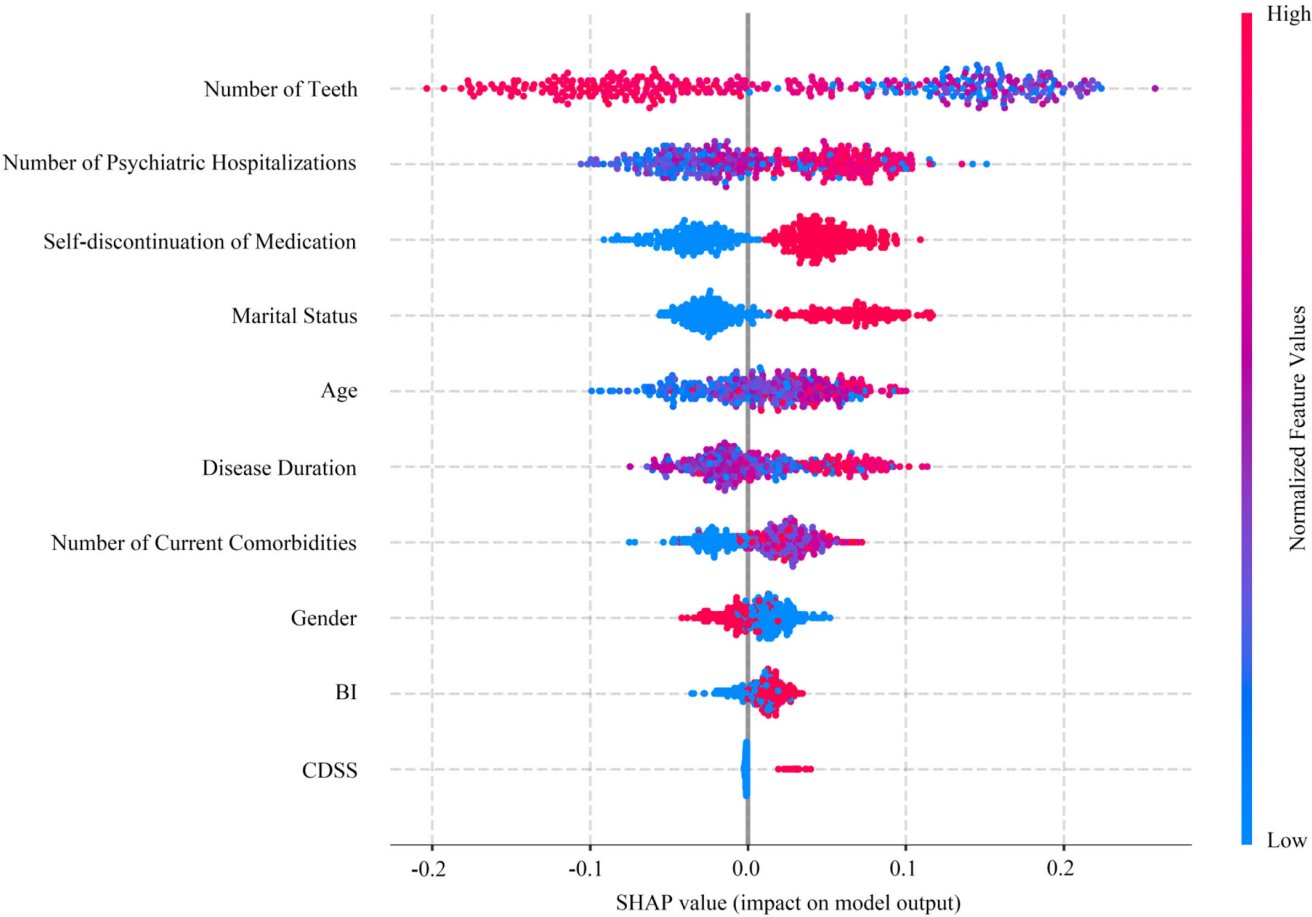
SHAP value distribution for key oral frailty-related features.

The SHAP-based feature importance swarm plot (**Figure 6**) quantifies the contribution of each variable to the risk of OF in patients with SZ. The horizontal axis represents SHAP values, indicating both the direction and magnitude of the impact on the OF score, while the vertical axis represents the independent variables. Color gradients reflect the magnitude of each feature’s value. The 10 independent variables are ranked in descending order of their influence on the OF score as follows: number of teeth > number of psychiatric hospitalizations > self-discontinuation of medication > marital status > age > disease duration > number of current comorbidities > gender > BI > CDSS.

## 4 Discussion

Long-term hospitalized SZ patients represent a high-risk group for OF however, research in this area remains limited and predominantly relies on traditional methodologies. This study employed a data-driven, two-stage feature selection approach combined with ML techniques to systematically investigate the prevalence and influencing factors of OF among long-term inpatients with SZ in southwestern China, providing an evidence-based foundation and technical support for clinical oral health management in psychiatric settings. The results indicate an OF prevalence of 69.3% in this study sample, which is significantly higher than the reported rates among community-dwelling older adults (32%) (38), stroke patients (47.7%) (39), and cancer patients (57.58%) (40). These findings further substantiate that long-term institutionalized SZ patients are at high risk for OF, underscoring the need for prioritized attention to oral health issues in structured care environments.

From a methodological perspective, the two-stage feature selection strategy developed in this study offers significant advantages. In the first stage, features associated with OF were preliminarily identified through the systematic combination of three categories, including nine feature selection methods and five classical classification models, with the Random Forest model emerging as the optimal classifier. In the second stage, by adjusting thresholds to integrate common features derived from different methodological categories, the AUC of the RF model was further increased to 0.779—an improvement of 1.96% over the best result achieved by any individual feature selection method. This approach mitigates the bias inherent in traditional single-method feature selection and results in an approximate 6.57% improvement compared to using no feature selection, thus enhancing both predictive performance and generalization capability. Furthermore, leveraging the SHAP framework enabled the interpretability of the ML model, quantifying each feature’s contribution to OF and revealing nonlinear relationships between features and the condition.

The Number of Teeth was the only variable retained by all nine feature selection methods in this study and serves as the primary influencing factor for oral function in hospitalized SZ patients, with tooth loss serving as a core indicator of OF impairment. Insufficient dentition directly compromises masticatory function, leading to malnutrition and further exacerbating the decline in oral function (41). Existing re-search has confirmed that the risk of complete tooth loss in individuals with severe mental illness is nearly three times higher than in the general population (42), while the tooth loss rate among elderly SZ patients reaches as high as 83.3% (43). The occurrence of tooth loss in SZ patients involves multiple contributing factors: first, di-minished motivation for oral self-care due to negative symptoms leads to prolonged accumulation of dental plaque (44). Second, this population exhibits a higher prevalence of dental caries and periodontal disease, both of which are primary causes of tooth loss (45). Finally, the anticholinergic side effects of antipsychotic medications reduce salivary flow, impairing oral cleansing and buffering capacity, thus accelerating the risks of caries, periodontal disease, and oral infections (46).

Number of Psychiatric Hospitalizations is positively associated with alterations in OF. Patients who experience more frequent hospital admissions often present with more severe clinical conditions, including greater impairments in cognitive function and self-care abilities (11, 44). Extended hospital stays can further reduce patients’ ability to maintain autonomous oral hygiene, thereby increasing their dependency on external care (47). Additionally, studies focusing on hospitalized populations indicate that nearly half of inpatients display OF abnormalities, which are significantly cor-related with oral Enterobacteriaceae colonization. These findings suggest that pro-longed hospitalization may lead to dysbiosis within the oral microbiome (48).

Marital Status is significantly associated with individual oral health, with the core mechanism rooted in the crucial role of spousal supervision of health behaviors and emotional support (49). A five-year cohort study conducted in Germany con-firmed that married individuals face a significantly lower risk of tooth loss compared to unmarried or widowed individuals (50). Meta-analyses further corroborate that Marital Status is positively associated with the utilization of oral healthcare services and the retention rate of teeth (51). In contrast, unmarried or widowed individuals experience increased oral health risks, primarily due to the absence of daily oral care monitoring and emotional support from a spouse (49). Furthermore, unmarried older adults are more vulnerable to negative emotional states such as depression and anxiety (52), which can further diminish their oral health awareness and accelerate the deterioration of their oral conditions (53).

Self-discontinuation of medication contributes to the development of OF by exacerbating disease fluctuations and impairing self-care capabilities. Patients with SZ require long-term, often lifelong, pharmacotherapy, with optimal medication adherence being crucial for therapeutic efficacy. However, poor adherence remains a widespread global issue; a meta-analysis indicates that more than half of patients with psychiatric disorders experience treatment interruptions (54, 55). Suboptimal medication adherence leads to disease progression, diminished treatment response, and a nearly fivefold increase in relapse risk (56). Disease recurrence not only reduces patients’ motivation to maintain oral hygiene but also exacerbates negative symptoms, further decreasing hygiene behaviors such as tooth brushing (57). These factors collectively accelerate plaque accumulation, dental caries, and periodontal disease progression, ultimately contributing to the development of OF.

Age is an independent risk factor for OF in hospitalized SZ patients. Age-related degenerative changes in the oral cavity manifest in several ways: First, aging leads to a decline in masticatory and swallowing functions, accompanied by gingival recession and a higher incidence of periodontal diseases, which directly compromise oral health and nutritional status (43). Second, aging reduces alkaline phosphatase activity in periodontal cells, impairing bone formation capacity, while gingival recession-induced root exposure further increases the risk of root caries (58, 59). In SZ patients, aging not only directly exacerbates the deterioration of oral physiological functions but also synergizes with disease-induced cognitive impairment and lack of motivation for self-care, further diminishing oral hygiene capacity. This ultimately leads to an imbalance in the oral microenvironment (20), emphasizing the critical impact of age on OF.

As public health awareness continues to grow, the overall oral health status of the general population has improved significantly. However, oral hygiene remains a significant unmet need in patients with SZ. Consequently, this population should be prioritized within dental healthcare services. Targeted interventions are required to enhance their oral health, which in turn will help preserve their quality of life, physical functionality, and social integration. Based on the results of this study, we propose several clinical interventions: First, patients with inadequate dentition should be prioritized through regular oral examinations, early treatment interventions, and, when necessary, prosthetic rehabilitation to restore masticatory function. Second, a structured oral care routine should be established for patients with frequent and pro-longed hospitalizations, with enhanced supervision from healthcare providers. Moreover, personalized oral health prevention and rehabilitation plans should be tailored for elderly patients to mitigate the decline in oral physiological functions. Special attention should be given to the social support needs of unmarried or widowed patients. Family-centered care strategies and community health initiatives can help alleviate the absence of spousal support. Additionally, improving medication adherence should be a key focus through health education, regular follow-ups, and strategies to reduce the self-discontinuation of medication, thus minimizing the adverse impact of disease fluctuations on oral health. Beyond these measures, patient education on oral health awareness and the development of individualized oral care plans that incorporate lifestyle modifications are critical for addressing the root causes of oral health deterioration.

Although this study presents methodological innovations and offers clinical translational value, several limitations should be considered for future research. First, the cross-sectional design precludes the ability to infer causality, as it only reveals associations among variables. Second, the sample was exclusively recruited from three psychiatric hospitals in Southwest China, which may limit the generalizability of the findings due to regional constraints. To improve external validity, future studies should aim to include multi-center and multi-regional study populations. Third, the assessment of OF relied solely on rating scales, lacking standardized objective biomarkers or instrumental measurements, which may affect the precision of the results. Future research should incorporate longitudinal designs, expand sample sizes, integrate data from multiple centers, and establish objective evaluation frameworks to validate and extend the findings.

## 5 Conclusion

This study employed a data-driven two-stage feature selection approach combined with ML techniques to determine that the prevalence of OF disorders among long-term hospitalized SZ patients in Southwest China is 69.3%. Five key influencing factors were identified: Number of Teeth, Number of Psychiatric Hospitalizations, Self-discontinuation of Medication, Marital Status, and Age. Using the two-stage feature selection strategy, the AUC of the OF prediction model was improved to 0.779, providing a reliable, evidence-based foundation for precise clinical interventions. Healthcare professionals are advised to pay particular attention to elderly patients with significant tooth loss, conduct regular oral examinations, and improve health education and medication adherence management. Furthermore, strengthening communication and support systems between patients and their families may improve oral health and contribute to overall patient recovery.

## Data Availability

The raw data supporting the conclusions of this article will be made available by the authors, without undue reservation.
